# Associations between the oxytocin receptor gene (*OXTR*) rs53576 polymorphism and emotional processing of social and nonsocial cues: an event-related potential (ERP) study

**DOI:** 10.1186/s40101-016-0125-3

**Published:** 2017-01-26

**Authors:** Damee Choi, Natsumi Minote, Shigeki Watanuki

**Affiliations:** 1 Faculty of Design, Kyushu University, Fukuoka, Japan; 2 Japan Society for the Promotion of Science, Tokyo, Japan; 3 National Institute of Advanced Industrial Science and Technology (AIST), Tsukuba, Japan; 4 Graduate School of Integrated Frontier Science, Kyushu University, Fukuoka, Japan

**Keywords:** OXTR, rs53576, ERP, Emotion, Social cue

## Abstract

**Background:**

Oxytocin receptor (*OXTR*) gene polymorphisms are related to individual differences in emotional processing of social cues. However, whether *OXTR* polymorphisms affect emotional processing of nonsocial cues remains unclear. The present study investigated the relationship between the *OXTR* rs53576 polymorphism and emotional processing of social cues and nonsocial cues.

**Methods:**

Event-related potentials were recorded from 88 male participants while images of humans and images of objects were presented as social cues and nonsocial cues, respectively.

**Results:**

First, the results showed that GG carriers of *OXTR* rs53576 showed more negative N1 (50–200 ms) than AA carriers in response to images of both humans and objects. Second, GG carriers showed more negative N2 (200–320 ms) than AA carriers in response to images of humans but not in response to images of objects. Third, GG carriers showed more negative N2 in response to images of humans than images of objects, whereas AA carriers showed the opposite pattern. Fourth, we observed no difference in late positive potential (600–1000 ms) to images of humans or objects that depended on the *OXTR* rs53576 polymorphism.

**Conclusions:**

These results suggest that the *OXTR* rs53576 polymorphism affects emotional processing of not only social cues but also nonsocial cues in the very early stage (reflected in N1); however, the data also suggest that the *OXTR* rs53576 polymorphism is related specifically to increased emotional processing of social cues in the middle stage (reflected in N2).

## Background

Social behaviors refer to “the reciprocal interactions of two or more animals and the resulting modification of the individual's action system” [1]. Oxytocin, a neuroactive hormone produced in the hypothalamus, is closely related to human social behaviors (reviewed in [2, 3]). For example, intranasal administration of oxytocin improves the ability to infer the mental state of others [4] and increases gazing toward the eye region of human faces [5]. Moreover, genetic variations in the gene for the oxytocin receptor (*OXTR*) are related to individual differences in responses to social cues. In particular, recent studies have found that rs53576, a single nucleotide polymorphism (SNP) in *OXTR*, is related to such individual differences. Behavioral studies have indicated that homozygous carriers of the G allele (GG carriers) show higher trait empathy [6, 7], prosocial behavior [8], trust behavior [9], and lower social loneliness [10] than those with the A allele (AA/GA carriers). These findings are also supported by physiological results indicating that GG carriers show facilitated brain activity to human faces [11, 12] and increased blood pressure and cortisol levels in response to social rejection [13] than AA/GA carriers. These studies suggested that the G allele of the *OXTR* rs53576 polymorphism is related to a higher sensitivity to social cues.

Some previous studies have examined whether administration of oxytocin affects responses to nonsocial cues [14, 15]. For instance, administration of oxytocin improves recognition memory for images including human figures (e.g., images of human faces) but not for images not including human figures (e.g., images of houses, art sculptures, and landscapes) [14]. Meanwhile, a recent study reported that administration of oxytocin enhances the social meaning of images of objects [15]. Although the relationship between the *OXTR* rs53576 polymorphism and the oxytocin level is still unclear (reviewed in [16]), a later study [15] suggests a possibility that genetic variations in *OXTR* may be related not only to individual differences in the response to social cues but also to differences in the response to nonsocial cues. However, the association between the rs53576 polymorphism and the response to nonsocial cues such as images of objects remains unclear, because most reported studies on this *OXTR* polymorphism have focused on responses to social cues such as human faces [11, 12] and social situations [6, 8, 9, 13]. Thus, the present study focused on a possible association between the *OXTR* rs53576 polymorphism and the response to nonsocial cues.

Event-related potential (ERP), the electroencephalogram (EEG) response to specific events such as presentation of emotional stimuli, reflects the time course of information processing in the brain due to its high temporal resolution (reviewed in [17, 18]). Studies of passive image viewing tasks have reported that mainly the following three ERP components are sensitive to emotional content; N1, N2/early posterior negativity (EPN), and late positive potential (LPP) [19–21]. N1 is a negative peak observed at around 130 ms, N2/ERN is a negativity observed at around 250 ms, and LPP is a sustained positivity that becomes evident 300 ms after stimulus onset. N2 is usually analyzed when a mastoid electrode reference is used, whereas ERN is usually analyzed when an average electrode reference is used (reviewed in [17]). The N1, N2/ERN, and LPP components are greater (more negative for N1 and N2/EPN; more positive for LPP) in response to unpleasant images than emotionally neutral images [19, 20, 22, 23]. Moreover, some studies suggested that N2 reflects an individual difference in response to social and nonsocial cues [21, 24, 25]. Taken together, the N1, N2/ERN, and LPP components are thought to reflect the time course of emotional processing.

In the present study, we aimed to investigate associations between the *OXTR* rs53576 polymorphism and the time course of emotional processing of social and nonsocial cues by measuring ERP responses. To do so, we analyzed the N1, N2, and LPP components of ERP responses from 88 young male individuals while images of humans and images of objects were presented as social cues and nonsocial cues, respectively. Given that previous studies showed a higher sensitivity to social cues in GG carriers [6–13], we hypothesized that GG carriers would show a greater ERP response (more negative N1 and N2; more positive LPP) than GA or AA carriers in response to images of humans. More importantly, if an *OXTR* polymorphism affects emotional processing of not only social cues, but also nonsocial cues, we would expect to see differences in ERP responses between the *OXTR* rs53576 genotype groups in response to images of objects.

## Methods

### Participants

Ninety-two male Japanese undergraduate or graduate students (age range 19–25 years) participated in this study. In the present study, we recruited only male participants because previous studies reported that males show clearer differences in brain structures [10] and emotional traits [11] according to the rs53576 polymorphism than females. Participants reported that they had no psychiatric disorders. Eighty-eight participants were included in the final analysis, because the quality of the EEG was poor for four participants (for specific details, refer to “ERP measurements and analysis” section). After receiving an explanation of the details of the study, participants provided written informed consent prior to participation.

### Genotyping

Genomic DNA was extracted from the saliva of participants using a Saliva DNA Isolation Kit (Norgen Biotek Corporation, Thorold, Ontario, Canada). Genotyping for the rs53576 polymorphism was then performed using TaqMan SNP Genotyping Assays (Applied Biosystems, Foster City, CA, USA). PCR amplification was carried out in a LightCycler Nano real-time PCR system (Roche Diagnostics, Mannheim, Germany). All samples were run twice, and they all provided consistent results. The genotype distribution (10 GG, 46 GA, and 32 AA carriers) was in Hardy-Weinberg equilibrium (*p* = 0.280) and was in line with previous studies showing that AA carriers are more common than GG carriers in Asian populations [26–29].

### Stimuli

A total of 270 images were selected from the International Affective Picture System (IAPS) [30]. The images consisted of three *content* categories: objects, humans, and animals. The images of objects and images of animals did not include figures of humans, whereas the images of humans included figures of more than one person. The images of animals were presented as fillers to buffer against possible habituation, and thus, the results from these were not included in the analysis. Within each c*ontent* category, the images were further subdivided into the following three *valence* categories: neutral, pleasant, and unpleasant. Examples of each category of images include a tissue box (neutral image of objects), flowers (pleasant image of objects), a dirty toilet (unpleasant image of objects), a man with an emotionally neutral face (neutral image of humans), a man with a baby (pleasant image of humans), an injured person (unpleasant image of humans), a fox (neutral image of animals), puppies (pleasant image of animals), and cockroaches (unpleasant image of animals). The category of pleasant images of humans did not include erotic images, because balancing arousal levels between pleasant images of humans and pleasant images of objects was difficult. Specific IAPS picture identification numbers [30] are presented in the Appendix.

### Procedures

Participants were seated approximately 80 cm from a screen (20-in. monitor). They were asked to focus on the screen and to look at the images presented. During EEG recording, three blocks of image presentations were shown. In each block, 90 images (10 images for each category) were presented three times for a total of 270 trials. In each trial, a white fixation cross was presented on a black screen for 500 ms, and then an image was presented for 1000 ms. The inter-trial interval was 1250–1750 ms, and the order of the trials was random. The images presented were different among the three blocks.

After EEG recording, each participant filled out a subjective assessment. They once again observed the images presented during the EEG recording and judged the valence and arousal of each image based on a 9-point Likert scale (for valence, “very pleasant” was assigned 9 points, whereas “very unpleasant” was assigned 1 point; for arousal, “very arousing” was assigned 9 points, whereas “very relaxing” was assigned 1 point).

### ERP measurements and analysis

The EEG was recorded using a 64-channel Geodesic Sensor Net (Electrical Geodesics, Inc., Eugene, OR, USA) based on the 10/20 system and was amplified by a high-input impedance (200 MΩ) amplifier (Net Amps 200 Amplifier, Electrical Geodesics, Inc.). During recording, EEG signals were recorded at electrode site Cz as a reference with a sampling frequency of 500 Hz. Electrode impedances were maintained below 50 kΩ.

After recording, EEG data were re-referenced offline to the average of the left and right mastoids and band-pass filtered with cutoffs of 0.1 and 30 Hz^1^ using EMSE software (Source Signal Imaging Inc., San Diego, CA, USA). The trials of image presentation were averaged for the time window between −200 to 1000 ms for each category of images. Trials including artifacts (eye blinks, muscle artifacts, and body movements) above ±100 μV were rejected. Four participants were excluded from the final analysis because their mean number of trials for each image category was less than 30. The mean number of trials (M) and the standard deviation (SD) of the final samples were the following: neutral images of objects, *M* = 70.2, SD = 14.2; pleasant images of objects, *M* = 70.2, SD = 14.9; unpleasant images of objects, *M* = 71.8, SD = 13.6; neutral images of humans, *M* = 70.9, SD =14.7; pleasant images of humans, *M* = 71.3, SD = 14.3; and unpleasant images of humans, *M* = 71.3, SD = 14.1.

We calculated three ERP components as follows: N1 (average amplitude between 50 and 200 ms), N2 (average amplitude between 200 and 320 ms), and LPP (average amplitude between 600 and 1000 ms). N1, N2, and LPP were all averaged from centro-parietal sites (CP1/2, P1/2, Pz, and POz) based on previous findings that the effect of emotional content on these ERP components is generally maximal in centro-parietal areas (for example, [19, 20] for N1 and LPP and [25, 31] for N2).

### Statistical analysis

For the ERP responses (N1, N2, and LPP) and the subjective ratings (valence and arousal ratings), we conducted a generalized linear mixed model with *OXTR* (GG vs. GA vs. AA), *content* (object vs. human), and *valence* (neutral vs. pleasant vs. unpleasant) as fixed factors, and *participant* (a personal code assigned to each subject) as a random factor. In case of significant main effects or interactions, a post hoc *t* test was performed with Bonferroni corrections. All statistical analyses were conducted using SPSS software (version 23, IBM, Chicago, IL, USA), and statistical significance was set at *p* < 0.05.

## Results

### ERP responses

**Fig. 1:**
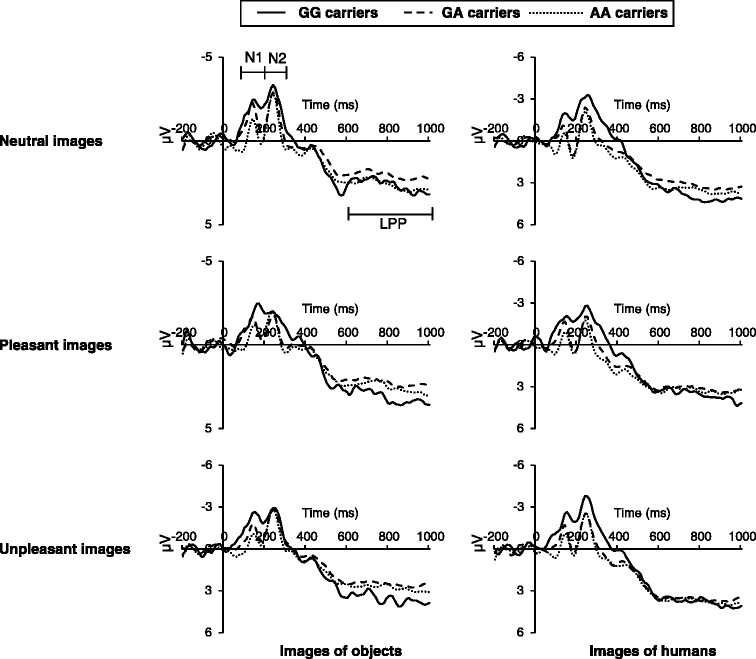
Grand averaged event-related potential (ERP) in centro-parietal sites for the oxytocin receptor (*OXTR*) genotype groups (GG vs. GA vs. AA carriers). *LPP* late positive potential

Grand averaged ERP waveforms in the centro-parietal area are shown in Fig. 1. A summary of the results of the generalized linear mixed model for the ERP responses is shown in Table 1, and the mean scores of the ERP responses are shown in Table 2.

**Table 1:** Results of the generalized linear mixed model for event-related potential (ERP) responses

		N1		N2		LPP	
Factor	df1, df2	*F*	*P*	*F*	*P*	*F*	*P*
OXTR	2, 510	3.40	*0.034*	0.96	0.383	0.67	0.517
Content	1, 510	25.67	*0.000*	0.35	0.554	51.07	*0.000*
Valence	2, 510	2.71	0.068	12.19	*0.000*	3.15	*0.044*
OXTR × content	2, 510	1.46	0.233	5.47	*0.004*	0.73	0.484
OXTR × valence	4, 510	0.82	0.515	0.19	0.945	0.33	0.855
Content × valence	2, 510	2.82	0.061	0.04	0.964	1.37	0.256
OXTR × content × valence	4, 510	0.69	0.601	0.73	0.574	0.52	0.721

Significant *p* values are in *italics*

*OXTR* oxytocin receptor, *LPP* late positive potential, *df* degrees of freedom

**Table 2:** Mean amplitude (μV) of event-related potential (ERP) responses

		All participants (*n* = 88)	GG carriers (*n* = 10)	GA carriers (*n* = 46)	AA carriers (*n* = 32)
N1					
Images of objects	Neutral	−0.76 (0.18)	−1.64 (0.53)	−1.01 (0.25)	−0.13 (0.30)
	Pleasant	−0.70 (0.18)	−1.44 (0.52)	−0.76 (0.24)	−0.38 (0.29)
	Unpleasant	−0.71 (0.21)	−1.76 (0.61)	−0.80 (0.28)	−0.26 (0.34)
Images of humans	Neutral	−0.01 (0.19)	−1.17 (0.56)	−0.06 (0.26)	0.43 (0.31)
	Pleasant	−0.35 (0.19)	−1.43 (0.55)	−0.43 (0.26)	0.11 (0.31)
	Unpleasant	−0.42 (0.18)	−1.67 (0.53)	−0.45 (0.25)	0.01 (0.29)
N2					
Images of objects	Neutral	−1.31 (0.27)	−2.04 (0.81)	−1.34 (0.38)	−1.04 (0.45)
	Pleasant	−0.81 (0.26)	−1.49 (0.79)	−0.74 (0.37)	−0.68 (0.44)
	Unpleasant	−1.51 (0.28)	−1.79 (0.85)	−1.61 (0.40)	−1.28 (0.48)
Images of humans	Neutral	−1.17 (0.29)	−2.59 (0.85)	−1.14 (0.40)	−0.77 (0.48)
	Pleasant	−0.79 (0.28)	−2.04 (0.84)	−0.85 (0.39)	−0.31 (0.47)
	Unpleasant	−1.30 (0.30)	−2.67 (0.90)	−1.20 (0.42)	−1.01 (0.50)
LPP					
Images of objects	Neutral	2.33 (0.21)	2.63 (0.63)	2.05 (0.29)	2.64 (0.35)
	Pleasant	2.46 (0.20)	3.14 (0.59)	2.25 (0.28)	2.54 (0.33)
	Unpleasant	2.74 (0.22)	3.57 (0.66)	2.51 (0.31)	2.80 (0.37)
Images of humans	Neutral	3.42 (0.23)	4.01 (0.69)	3.20 (0.32)	3.56 (0.39)
	Pleasant	3.26 (0.22)	3.59 (0.65)	3.16 (0.30)	3.29 (0.37)
	Unpleasant	3.69 (0.24)	3.82 (0.71)	3.60 (0.33)	3.76 (0.40)

Standard error in *parentheses*

*LPP* late positive potential

**Fig. 2:**
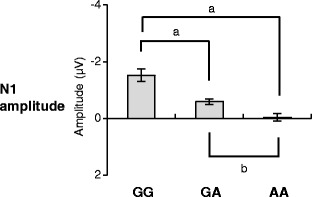
Mean amplitude of N1 of event-related potential (ERP) for the oxytocin receptor (*OXTR*) genotype groups (GG vs. GA vs. AA carriers). Data were collapsed across valence and content of images. Error bars indicate standard error. (*a*) *p* < 0.001 (critical *p* value = 0.017); (*b*) *p* = 0.001 (critical *p* value = 0.017)

For N1, we observed a significant main effect of *OXTR* (Table 1). Post hoc analysis (independent samples *t* test, critical *p* value = 0.017 for three comparisons) revealed that GG carriers showed significantly more negative N1 than GA (*t*(334) = −3.90, *p* < 0.001) and AA carriers (*t*(250) = −5.51, *p* < 0.001) and that GA carriers showed significantly more negative N1 than AA carriers (*t*(466) = −3.35, *p* = 0.001) (Fig. 2). We also observed a significant main effect of *content* for N1 (Table 1), indicating that N1 is significantly more negative in response to images of objects (*M* = −0.72 μV, standard error (SE) = 0.11) than images of humans (*M* = −0.26 μV, SE = 0.11).

**Fig. 3:**
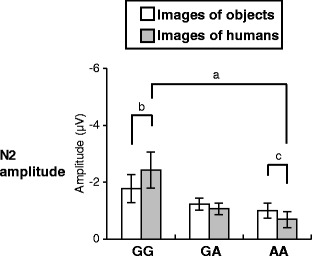
Mean amplitude of N2 of event-related potential (ERP) for the oxytocin receptor (*OXTR*) genotype groups (GG vs. GA vs. AA carriers). Data were collapsed across valence of images. Error bars indicate standard error. (*a*) *p* = 0.016 (critical *p* value = 0.017); (*b*) *p* = 0.040 (critical *p* value = 0.050); (*c*) *p* = 0.031 (critical *p* value = 0.050)

For N2, we observed a reliable interaction of *OXTR* × *content* (Table 1). As shown in Fig. 3, post hoc analysis between *OXTR* within each *content* (independent samples *t* test, critical *p* value = 0.017 for three comparisons) revealed that GG carriers showed significantly more negative N2 than AA carriers in response to images of humans (*t*(41.0) = −2.50, *p* = 0.016), whereas we found no significant difference in N2 among GG, GA, and AA carriers in response to images of objects (all *p* > 0.017) (Fig. 3). Post hoc analysis between *content* within each *OXTR* (paired samples *t* test) was also conducted; GG carriers showed significantly more negative N2 in response to images of humans than images of objects (*t*(29) = 2.15, *p* = 0.040). GA carriers did not show significant differences in N2 in response to images of objects compared to images of humans (*t*(137) = −1.40, *p* = 0.165), and AA carriers showed significantly more negative N2 in response to images of objects than images of humans (*t*(95) = −2.19, *p* = 0.031) (Fig. 3). We also observed a main effect of *valence* (Table 1) for N2. Post hoc analysis (paired samples *t* test, critical *p* value = 0.017 for three comparisons) indicated that N2 was significantly more negative in response to neutral images (*M* = −1.24 μV, SE = 0.20) and unpleasant images (*M* = −1.41 μV, SE = 0.21) than pleasant images (*M* = −0.80 μV, SE = 0.19) (neutral vs. pleasant: *t*(175) = −4.63; unpleasant vs. pleasant: *t*(175) = 6.15; all *p* < 0.001).

For LPP, we found no main effect of *OTXR*, and related interactions were not significant (Table 1). We observed a significant main effect of *content* for LPP (Table 1), indicating that LPP was significantly more positive in response to images of humans (*M* = 3.46 μV, SE = 0.13) than images of objects (*M* = 2.51 μV, SE = 0.12). We also observed a significant main effect of *valence* for LPP (Table 1). Post hoc analysis (paired samples *t* test, critical *p* value = 0.017 for three comparisons) revealed that LPP was significantly more positive in response to unpleasant images (*M* = 3.21 μV, SE = 0.17) than neutral images (*M* = 2.88 μV, SE = 0.16) and pleasant images (*M* = 2.86 μV, SE = 0.15) (neutral vs. unpleasant: *t*(175) = −2.98, *p* = 0.003; pleasant vs. unpleasant: *t*(175) = −2.74, *p* = 0.007).

### Subjective ratings

Table 3 summarizes the results of the generalized linear mixed model for the subjective ratings, and Table 4 shows the mean scores of subjective ratings.

**Table 3:** Results of the generalized linear mixed model for subjective ratings

		Valence		Arousal	
Factor	df1, df2	*F*	*P*	*F*	*P*
OXTR	2, 510	0.14	0.867	0.136	0.873
Content	1, 510	7.17	*0.008*	85.00	*0.000*
Valence	2, 510	333.67	*0.000*	89.63	*0.000*
OXTR × content	2, 510	0.17	0.844	3.97	*0.019*
OXTR × valence	4, 510	2.56	*0.038*	2.51	*0.041*
Content × valence	2, 510	3.18	*0.042*	0.28	0.756
OXTR × content × valence	4, 510	0.32	0.863	0.25	0.912

Significant *p* values are in *italics*

*OXTR* oxytocin receptor, *df(h,e)* degrees of freedom

**Table 4:** Mean scores of subjective ratings

		All participants (*n* = 88)	GG carriers (*n* = 10)	GA carriers (*n* = 46)	AA carriers (*n* = 32)
*Valence*					
Images of objects	Neutral	5.05 (0.03)	4.99 (0.09)	5.09 (0.04)	5.03 (0.05)
	Pleasant	5.81 (0.06)	5.64 (0.17)	5.78 (0.08)	5.91 (0.10)
	Unpleasant	3.69 (0.07)	4.01 (0.20)	3.67 (0.09)	3.64 (0.11)
Images of humans	Neutral	5.36 (0.05)	5.26 (0.16)	5.33 (0.07)	5.44 (0.09)
	Pleasant	6.01 (0.07)	5.75 (0.22)	6.01 (0.10)	6.09 (0.12)
	Unpleasant	3.65 (0.08)	4.02 (0.25)	3.59 (0.12)	3.62 (0.14)
*Arousal*					
Images of objects	Neutral	4.07 (0.12)	4.10 (0.35)	4.14 (0.16)	3.95 (0.20)
	Pleasant	4.48 (0.10)	4.39 (0.31)	4.51 (0.14)	4.48 (0.17)
	Unpleasant	5.34 (0.09)	5.05 (0.26)	5.50 (0.12)	5.21 (0.14)
Images of humans	Neutral	4.64 (0.10)	4.64 (0.29)	4.57 (0.13)	4.75 (0.16)
	Pleasant	5.14 (0.12)	5.04 (0.35)	4.96 (0.16)	5.42 (0.20)
	Unpleasant	6.01 (0.10)	5.65 (0.28)	6.11 (0.13)	5.98 (0.16)

Standard error in *parentheses*

Valence: 1 = “very unpleasant,” 9 = “very pleasant”; arousal: 1 = “very relaxing,” 9 = “very arousing”

For valence ratings, we observed a reliable interaction of *OXTR* × *valence* (Table 3). Post hoc analysis between *OXTR* within each *valence* (independent samples *t* test, critical *p* value = 0.017 for three comparisons) was conducted; however, we found no significant differences in valence rating among GG, GA, and AA carriers for neutral, pleasant, or unpleasant images (all *p* > 0.017). We identified main effects of *content* and *valence*, and a reliable interaction of *content* × *valence* for valence ratings (Table 3). Post hoc analysis between *valence* within each *content* (paired samples *t* test, critical *p* value = 0.017 for three comparisons) indicated that participants reported pleasant images to be more pleasant than neutral images and unpleasant images to be more unpleasant than neutral images for both images of objects and images of humans (all *p* < 0.001).

For arousal ratings, we observed a reliable interaction of *OXTR* × *content* (Table 3). Post hoc analysis between *OXTR* within each *content* (independent samples *t* test, critical *p* value = 0.017 for three comparisons) was conducted; however, we found no significant differences in the arousal rating among GG, GA, and AA carriers for either images of objects or images of humans (all *p* > 0.017). Post hoc analysis between *content* within each *OXTR* (paired samples *t* test) was also conducted; GG, GA, and AA carriers all reported that images of humans were significantly more arousing than images of objects (all *p* < 0.001). We also observed a reliable interaction of *OXTR* × *valence* for arousal rating (Table 3). Post hoc analysis between *OXTR* within each *valence* (independent samples *t* test, critical *p* value = 0.017 for three comparisons) was conducted; however, we found no significant differences in arousal rating among GG, GA, and AA carriers for neutral, pleasant, or unpleasant images (all *p* > 0.017). The main effect of *content* was also significant for arousal ratings (Table 3), indicating that participants reported images of humans (*M* = 5.26, SE = 0.07) to be significantly more arousing than images of objects (*M* = 4.63, SE = 0.67). The main effect of *valence* was also significant for arousal ratings (Table 3). Post hoc analysis (paired samples *t* test, critical *p* value = 0.017 for three comparisons) revealed that participants reported unpleasant images (*M* = 5.68, SE = 0.07) to be more arousing than pleasant images (*M* = 4.81, SE = 0.08) and pleasant images to be more arousing than neutral images (*M* = 4.36, SE = 0.08) (all *p* < 0.001).

## Discussion

The present study investigated whether the *OXTR* rs53576 polymorphism affects emotional processing of social and nonsocial cues. To do so, we compared the ERP responses (N1, N2, and LPP) evoked by images of humans (social cues) and objects (nonsocial cues) among *OXTR* rs53576 genotype groups.

### Association between the OXTR rs53576 polymorphism and the ERP responses

In the present study, N1 was more negative in GG carriers of *OXTR* rs53576 than AA carriers, and intermediate in GA carriers, regardless of the response to images of objects or humans. Previous studies have reported that N1 is sensitive to highly emotional stimuli [19, 20]. Although the effect of valence of images on N1 did not reach a significant level in the present study (Table 1), the present result suggests that GG carriers of *OXTR* rs53576 show enhanced emotional processing compared to GA and AA carriers in the very early stage (50–200 ms) in response to both social and nonsocial cues. Regarding social cues, this result supports previous findings of a higher sensitivity to social cues in GG carries than in AA/GA carriers [6–13]. In particular, the present result replicated the previous ERP study [12] showing that the effect *OXTR* rs53576 on emotional processing of social cues occurs from the very early stage (reflected in N1). Moreover, given that Peltola et al. [12] adapted a task to discriminate facial expressions and the present study adapted a passive picture viewing task, we suggest that associations between *OXTR* rs53576 and early processing of social cues is evident regardless of whether active attention to social cues is required or not.

Regarding nonsocial cues, the present result of N1 provides new evidence that the *OXTR* rs53576 polymorphism affects emotional processing of nonsocial cues. One previous study [15] found that administration of oxytocin improves the subjective rating of emotional intensity of images of objects including the social context (touch between objects) but not the subjective rating of emotional intensity of images of objects not including social context (no touch between objects). In the present study, although images of objects did not include specific social context such as touching, N1 for images of objects was different depending on the *OXTR* rs53576 polymorphism. One study reported no difference in the oxytocin level between GG/GA carriers and AA carriers of the *OXTR* rs53576 polymorphism [32]; however, the relationship between the rs53576 polymorphism and the oxytocin level is still unclear (reviewed in [16]). Thus, interpretation of the mechanism of how the rs53576 polymorphism modulates responses to nonsocial cues remains difficult. Future studies are needed to examine the relationships among the *OXTR* rs53576 polymorphism, oxytocin levels, and emotional processing of nonsocial cues.

For N2, we found that GG carriers of *OXTR* rs53576 showed more negative N2 than AA carriers in response to images of humans but not in response to images of objects. Moreover, GG carriers showed a greater N2 in response to images of humans than to images of objects, whereas AA carriers showed a greater N2 in response to images of objects than to images of humans. In the present study, N2 was more negative in response to negative images than to pleasant images, supporting previous results showing that N2 is sensitive to highly emotional stimuli [19, 20, 22]. Thus, we suggest that GG carriers and AA carriers of *OXTR* rs53576 show opposite patterns regarding emotional processing of social cues and nonsocial cues in the middle stage (200–320 ms); GG carriers may show enhanced emotional processing of social cues compared to nonsocial cues, whereas AA carriers may show enhanced emotional processing of nonsocial cues compared to social cues. Similarly, Proverbio et al. [24, 25] reported that N2 is more negative in response to images portraying persons than images portraying landscapes in women, but not in men, suggesting that this result is caused by a greater interest in social stimuli for women compared with men. From this interpretation, the association between *OXTR* rs53576 and N2 that is shown in the present study may also be explained by the idea that GG carriers have a greater interest in social cues than AA carriers. This supports the previous findings of a higher sensitivity to social cues in GG carries than AA/GA carriers [6–13].

LPP did not show any differences related to *OXTR* rs53576, unlike N1 and N2. Thus, the present results for LPP suggest that *OXTR* rs53576 does not affect the processing of emotional stimuli in the relatively late stage (600–1000 ms), regardless of the existence of social content in the stimuli. The observation of no association between *OXTR* rs53576 on late processing of social cues is in line with the previous result by Peltola et al. [12], who reported an association between *OXTR* rs53576 and ERP responses to human faces in N1, but not in LPP. Taken together, we suggest that the association between *OXTR* rs53576 and emotional processing may be more evident in the early stage than in the late stage. However, because the present study adapted a passive image viewing task and the previous study adapted a relatively simple cognitive task [12], future studies are needed to examine possible effects of *OXTR* rs53576 on emotional processing in the late stage during complex cognitive tasks such as the memory task that was adopted in Rimmele et al. [14].

### Association between the OXTR rs53576 polymorphism and the subjective ratings

We found no difference in subjective ratings for valence and arousal of images among GG, GA, and AA carriers of the *OXTR* rs53576 polymorphism, although the results of our ERP response indicated differences in emotional processing of social cues and nonsocial cues among the different carriers of the *OXTR* rs53576 polymorphism. Although some studies (for example, [33]) suggest that physiological responses are more direct indices of responses than subjective ratings, future studies are needed to examine the association between the *OXTR* rs53576 polymorphism and subjective rating of nonsocial cues.

### Implications for anthropology

The distribution of the *OXTR* rs53576 genotype is different between Asians and European Americans; more GG carriers than AA carriers are found among European Americans, whereas more AA carriers than GG carriers are found among Asians [26, 27, 34, 35]. The present study also replicated previous results showing that AA carriers are more common than GG carriers in Asian populations (10 GG, 46 GA, and 32 AA carriers). This difference in the distribution of *OXTR* rs53576 carriers seems to be related to cultural differences in human behavior and emotion. For instance, one study [34] found that the frequency of the A allele of *OXTR* rs53576 is related to collectivistic cultural values. Another study on the distribution of *OXTR* rs53576 in Africa, Asia, and South Europe [35] suggested that the A allele of *OXTR* rs53576 may be related to favoritism toward sons. As one factor affecting the interaction between genes and culture, future studies should investigate the evolutionary route that resulted in the difference in the distribution of *OXTR* rs53576 among regions.

### Limitations and future directions

The present study has some important limitations. First, our sample size was small (*n* = 88) compared with most previous studies on associations between genotype and behavior or brain activity (for example, *N* = 94 [12], *N* = 108 [9], *N* = 179 [7], *N* = 228 [11], *N* = 285 [10]). Furthermore, the number of GG carriers in the present study was small (*N* = 10). As mentioned above, the distribution of the *OXTR* rs53576 genotype is different between Asians and European Americans. For this reason, several previous studies combined GA and AA carriers in their analyses [6, 7, 12], whereas other previous studies combined GG and GA carriers [26, 29, 32, 36]. However, other previous studies did not combine genotypes and compared GG, GA, and AA carriers [11, 13, 27, 28]. These different methods of grouping participants make the comparison of the findings between the present study and previous studies somewhat difficult.

Second, we examined only one *OXTR* SNP—rs53576. Although rs53576 is the most widely investigated SNP regarding the association between *OXTR* polymorphisms and variations in social behavior, previous studies have shown that other *OXTR* SNPs, such as rs7632287 [37], rs401015 [38], and rs2254298 [10, 35], also affect human social behavior. For instance, the *OXTR* rs7632287 polymorphism is related to individual differences in pair-bonding behavior [37]. Thus, future studies need to be conducted with analyses of other *OXTR* SNPs.

Third, our participants were all male. Thus, we could not investigate the possible interaction among gender, the *OXTR* rs53576 polymorphism, and emotional processing that has been suggested in previous studies [10, 11]. Future studies adopting the same methods as the present study with the inclusion of female participants are necessary.

## Conclusion

The present study investigated an association between the *OXTR* rs53576 polymorphism and the time course of emotional processing of social and nonsocial cues by measuring the ERP response. From the present results, we suggest that the *OXTR* rs53576 polymorphism affects emotional processing of not only social cues but also nonsocial cues in the very early stage (before 200 ms); however, we also suggest that the *OXTR* rs53576 polymorphism is related specifically to increased emotional processing of social cues in the middle stage (200–320 ms).

## Appendix

International Affective Picture System (IAPS) picture identification numbers:

Neutral images of objects: 5535, 6150, 6900, 7000, 7002, 7003, 7004, 7006, 7009, 7010, 7012, 7014, 7016, 7017, 7025, 7032, 7034, 7035, 7038, 7041, 7043, 7045, 7055, 7056, 7059, 7077, 7090, 7150, 7950, 9422.

Pleasant images of objects: 5000, 5001, 5010, 5020, 5030, 5040, 5200, 5202, 5450, 5471, 5480, 5800, 5890, 6910, 7042, 7053, 7058, 7061, 7095, 7096, 7100, 7140, 7900, 8162, 8170, 8325, 8501, 8502, 8510, 8531.

Unpleasant images of objects: 6020, 6930, 7013, 7023, 7046, 7054, 7078, 7135, 7137, 9080, 9090, 9110, 9290, 9295, 9300, 9320, 9440, 9480, 9600, 9610, 9611, 9620, 9621, 9622, 9630, 9830, 9904, 9909, 9911, 9912.

Neutral images of humans: 2191, 2215, 2221, 2273, 2384, 2397, 2410, 2442, 2445, 2484, 2485, 2487, 2570, 2575, 2580, 2745.1, 2770, 4520, 8010, 8065, 8121, 8160, 8191, 8250, 8251, 8260, 8320, 8341, 8465, 9210.

Pleasant images of humans: 2080, 2150, 2331, 2791, 4542, 5470, 5626, 5628, 5629, 5836, 8001, 8021, 8031, 8034, 8041, 8130, 8158, 8161, 8163, 8186, 8193, 8200, 8208, 8220, 8340, 8350, 8370, 8400, 8467, 8492.

Unpleasant images of humans: 2694, 2750, 6021, 6190, 6211, 6212, 6213, 6300, 6312, 6370, 652, 6831, 6832, 8320, 8480, 8485, 9002,9007, 9230, 9270, 9404, 9413, 9414, 9420, 9425, 9426, 9427, 9428, 9635, 9810.

Neutral images of animals: 1026, 1030, 1040, 1114, 1121, 1122, 1240, 1302, 1313, 1321, 1333, 1350, 1390, 1505, 1560, 1595, 1616, 1640, 1645, 1661, 1670, 1675, 1726, 1820, 1850, 1908, 1931, 1935, 1945, 1947.

Pleasant images of animals: 1410, 1419, 1440, 1441, 1460, 1463, 1500, 1510, 1540, 1590, 1600, 1603, 1604, 1605, 1610, 1620, 1630, 1640, 1650, 1660, 1710, 1720, 1721, 1722, 1740, 1750, 1810, 1812, 1900, 1920.

Unpleasant images of animals: 1050, 1051, 1052, 1090, 1110, 1113, 1120, 1200, 1202, 1205, 1220, 1270, 1271, 1274, 1300, 1301, 1525, 1930, 1932, 9180, 9181, 9182, 9183, 9184, 9185, 9186, 9187, 9560, 9561, 9571.

## Abbreviations

EEG: Electroencephalogram
EPN: Early posterior negativity
ERP: Event-related potential
IAPS: International affective picture system
LPP: Late positive potential
M: Mean
*OXTR*: Oxytocin receptor gene
SD: Standard deviation
SE: Standard error
SNP: Single nucleotide polymorphism

## Footnotes

1 We note that 0.1-Hz high-pass filter may not be appropriate to calculate LPP (please refer to [39]).
